# Trends in Keratoplasty Procedures During 2 Decades in a Major Tertiary Referral Center in Finland: 1995 to 2015

**DOI:** 10.1097/ICO.0000000000002990

**Published:** 2022-01-25

**Authors:** Olli Ala-Fossi, Kari Krootila, Tero T. Kivelä

**Affiliations:** * Department of Ophthalmology, University of Helsinki and Helsinki University Hospital, Helsinki, Finland; and; † Department of Ophthalmology, Helsinki University Hospital and University of Helsinki, Helsinki, Finland.

**Keywords:** keratoplasty, primary graft, regraft, PKP, DALK, ALTK, DSAEK, age-adjusted frequency

## Abstract

The purpose of this study was to analyze trends in number, age-adjusted frequency, and type of keratoplasty in a major tertiary referral center, relative to patient and graft characteristics.

**Methods:**

A retrospective registry study of 1574 patients who in 1995 to 2015 underwent keratoplasty in the Helsinki University Eye Hospital (HUEH). Graft type and sequence, patient characteristics, and date of surgery were recorded. Main outcome measures were annual number, type, and age-adjusted frequency of keratoplasty; patient and graft characteristics; graft procurement; and national population-adjusted frequency of keratoplasty.

**Results:**

In HUEH, from 1995 to 2015, a total of 2191 keratoplasties were performed with 48% of the grafts procured intramurally; 76% were primary and 24% regrafts. The age-adjusted frequency of primary penetrating keratoplasty decreased by 52% from 0.96 to 0.46 per 100,000. The corresponding frequency of primary Descemet stripping automated endothelial keratoplasty increased by 367% from 0.3 to 1.4 after 2006, finally accounting for 68% of primary grafts. Men underwent primary penetrating keratoplasty (median 48 vs. 67 yrs, *P* = 0.0001) and anterior lamellar keratoplasty (median 37 vs. 46 yrs, *P* = 0.0015) at a younger age than women. Interval to the first regraft was comparable between sexes (median 2.2 vs. 1.9 yrs, respectively, *P* = 0.17). The national median population-adjusted frequency of keratoplasties was 3.2 per 100,000 from 2009 to 2015, and HUEH accounted for a median of 69% of them.

**Conclusions:**

The increased frequency of keratoplasty in HUEH resulted from rapid adoption of Descemet stripping automated endothelial keratoplasty after 2006 and was facilitated by centralizing graft procurement to HUEH and the National Cell and Tissue Center Regea.

## Abstract 2

Supplemental Digital Content is Available in the Text.

The first successful human PKP was performed in 1905 by Zirm^1^ in Austria. Half a century later, Tillet introduced posterior lamellar keratoplasty,^2^ but PKP remained the mainstay in corneal transplantation until the end of the century. In recent decades, after the rapid development of microsurgical techniques, interest in anterior lamellar keratoplasty techniques (ALTK/DALK) resurfaced,^3–5^ and a novel technique for posterior lamellar keratoplasty was introduced in 1998,^6^ quickly followed by Descemet stripping endothelial keratoplasty in 2005,^7^ refined with the use of an automated microkeratome resulting as Descemet stripping automated endothelial keratoplasty (DSAEK) 1 year later.^8^ Descemet membrane endothelial keratoplasty (DMEK) was introduced concurrently in 2006^9^ and then began to compete for dominance.

These global advances in keratoplasty procedures have been translated to practice in different ways in individual centers that perform keratoplasty, although the general trend can be summarized as a marked decrease in the frequency of PKP in favor of anterior lamellar keratoplasties and endothelial keratoplasties (EKs).^10–20^ We set out to analyze the trends in the number and types of keratoplasty procedures not only in absolute numbers but also especially in relation to the changing demographics in industrialized countries, focusing on a major tertiary referral center in Northern Europe during 2 decades of rapid change, from 1995 to 2015.

## METHODS

### Aims of the Study

The primary aim was to analyze the number, annual age-adjusted frequency, and type of primary and all keratoplasty procedures performed from 1995 to 2015 in Helsinki University Eye Hospital (HUEH) and related patient characteristics. The secondary aims were to analyze the data in view of the procurement of corneal grafts in HUEH from 2000 to 2015 and the population-adjusted frequency of keratoplasties in Finland from 2009 to 2015.

### Study Design

Eligible for this retrospective registry study were patients who underwent any type of keratoplasty in the HUEH from 1995 to 2015. Patients were identified by cross-referencing the files of its Eye Bank, founded in 1956, and Ophthalmic Pathology Laboratory, founded in 1962. This study was approved by the Ethics Board of the Health Care District of Helsinki and Uusimaa.

### Data Collection

In HUEH, from 1995 to 2015, patient-related and graft-related factors were manually drawn from patient charts and histopathology reports. Patient-related factors recorded were birth date, sex, and relevant clinical history. Graft-related factors were date of each keratoplasty procedure, the type of graft, and the sequence of the graft.

**FIGURE 1.**
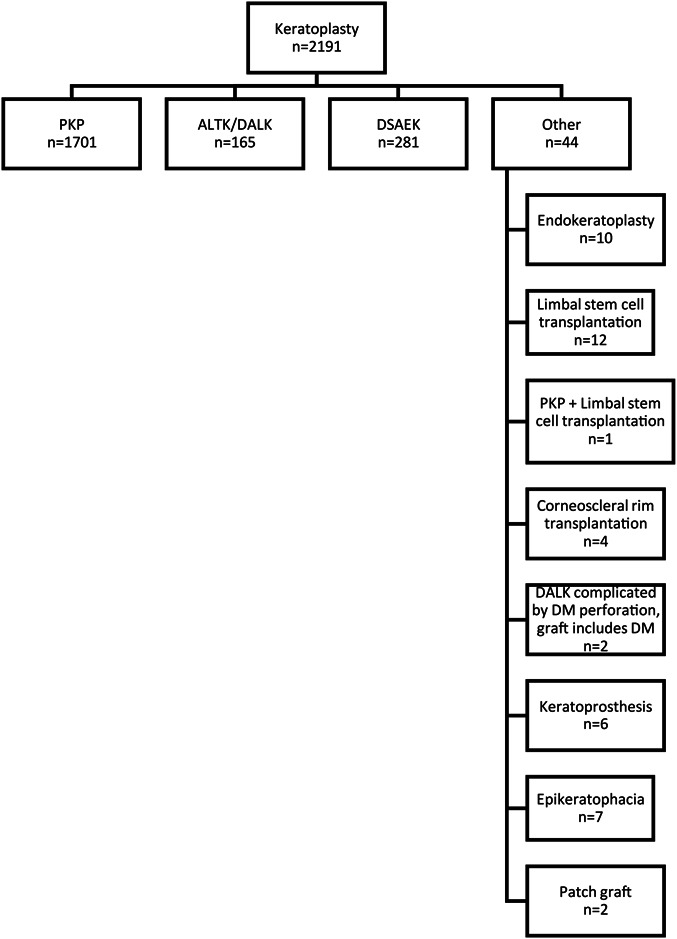
Type and number of keratoplasty procedures performed in Helsinki University Eye Hospital from 1995 to 2015. PKP, penetrating keratoplasty; ALTK/DALK, automated lamellar therapeutic keratoplasty/deep anterior lamellar keratoplasty; DSAEK, Descemet stripping automated endothelial keratoplasty; DM, Descemet membrane.

Keratoplasty procedures were categorized as PKP, including intended DALK procedures complicated by Descemet membrane perforation and converted to PKP, ALTK/DALK, DSAEK, and others (Fig. 1). The latter group included endokeratoplasty, limbal stem cell transplantation with or without PKP, corneoscleral rim transplantation, intended DALK-type procedures when a full-thickness graft was transplanted without removing Descemet membrane after it perforated, nonpenetrating patch grafts, keratoprosthesis, and epikeratophakia. DMEK was introduced in HUEH in 2017 and was not performed during the study period.

We also collected the total number of keratoplasties performed in Finland from 2009 to 2015 through personal communication with the other centers. HUEH Eye Bank provided the numbers of cornea donors from 2000 to 2015, including the origin of the donor graft, either self-acquired or purchased at cost.

### Statistical Analysis

Data were entered in a Microsoft Access database (Microsoft, Seattle, WA) and analyzed using Stata 15.0 software (StataCorp, College Station, TX). Relative frequency of keratoplasty procedures by year of grafting was calculated. Locally weighted scatterplot smoothing was used to describe trends in the annual crude number and the age-adjusted and population-adjusted frequency of primary and all keratoplasties. Cumulative frequency plots were used to compare age at the time of primary keratoplasty by sex and graft type, age at the first regraft by sex, and time to the first regraft by sex.

For calculating the age-adjusted frequency of both primary and total keratoplasties in HUEH, to allow comparison over time given changing population structure, population data were obtained from Statistics Finland. The standard population used was European Union Standard population.^21^ For calculating the national population-adjusted frequency of keratoplasty, to allow comparison over time given changing total population size, the data included the total number of keratoplasties in Finland from 2009 to 2015, irrespective of the type of graft performed and patient characteristics. The Kruskal–Wallis test was used to compare continuous variables by sex and graft type. The level of statistical significance was set at 0.05. All tests were 2-sided.

## RESULTS

### Keratoplasties Performed in HUEH

**FIGURE 2.**
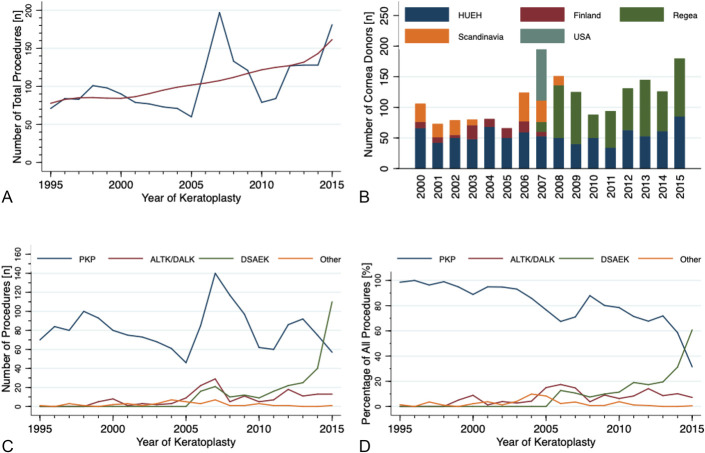
The annual number of all keratoplasty procedures (A), the annual number of cornea donors by eye bank (B), and keratoplasties divided by type of procedure (C), as well as their relative frequencies (D), in 1995-2015 in Helsinki University Eye Hospital (HUEH). The red line in (A) is locally weighted scatterplot smoothing. PKP, penetrating keratoplasty; ALTK/DALK, automated lamellar therapeutic keratoplasty/deep anterior lamellar keratoplasty; DSAEK, Descemet stripping automated endothelial keratoplasty; Other, other type of keratoplasty (see Figure 1).

From 1995 to 2015, altogether 2191 keratoplasties in 1837 eyes of 1574 patients were performed, of whom 265 (17%) received 1 or more grafts in both eyes during the study period (see Supplemental Table 1, Supplemental Digital Content 1, http://links.lww.com/ICO/B363). The median annual number of keratoplasty procedures was 90 (range, 60–197; mean 104). The lowest number of keratoplasties was in 2005 and the highest numbers in 2007 and 2015, respectively (Fig. 2A).

### Types of Corneal Grafts in HUEH

Of all procedures, 78% were PKP, 8% ALTK/DALK, 13% DSAEK, and 2% other procedures (see Supplemental Table 1, Supplemental Digital Content 1, http://links.lww.com/ICO/B363). DALK was introduced in 1999, ALTK in 2004, and DSAEK in 2006. After an initial slow increase, beginning from 2013, DSAEK rapidly became the most frequent keratoplasty procedure in 2015 (Figs. 2C, D). The proportion of PKP fell permanently below 90% in 2004, below 80% in 2010, and below 40% in 2015 (Fig. 2D).

### Primary Grafts in HUEH

Of all procedures, 76% were primary grafts. Of these, 73% were PKP, 9% ALTK/DALK, 16% DSAEK, and 2% other procedures (see Supplemental Table 1, Supplemental Digital Content 1, http://links.lww.com/ICO/B363). An increasing trend in the number of primary keratoplasty procedures was observed from 1995 (65 procedures) to 2015 (148 procedures). The percentage of primary PKP fell from 98% in 1995 to 75% in 2009, and to 24% in 2015. The percentage of primary DSAEK increased from 17% in 2006 to 68% in 2015 (see Supplemental Figs. 1B, C, Supplemental Digital Content 2, http://links.lww.com/ICO/B360).

### Regrafts in HUEH

Of all procedures, 24% were regrafts of which most were PKP (93%), and only 2% were ALTK/DALK, 3% DSAEK, and 2% other procedures; 67%, 19%, 8%, and 6% of them were first, second, third, and fourth or later regrafts, respectively (see Supplemental Table 1 and Supplemental Fig. 2, Supplemental Digital Content 3, 1, http://links.lww.com/ICO/B361, http://links.lww.com/ICO/B363). Regarding patients who received their primary graft during the study interval, 13% also received 1 or more regrafts. Supplemental Figure 3, Supplemental Digital Content 4, http://links.lww.com/ICO/B362 displays the type of regraft by the type of primary graft in the eyes, in which the primary and the first regraft were performed in 1995 to 2015.

### Age-Adjusted Frequency of Keratoplasty in HUEH

**FIGURE 3.**
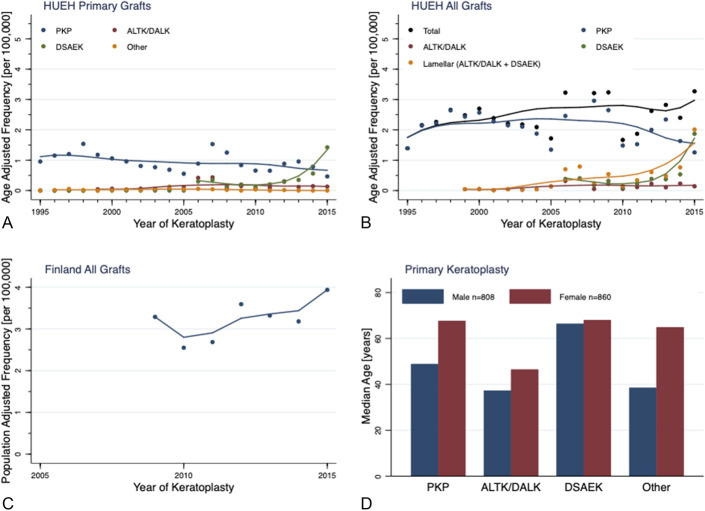
Age-adjusted frequency of primary keratoplasty procedures in HUEH by graft type (A) and all keratoplasties in HUEH (B) from 1995 to 2015 per 100,000. Population-adjusted frequency of all keratoplasties in Finland from 2009 to 2015 per 100,000 (C) and the median age at primary keratoplasty by sex and graft type (D). The lines in figure (A–C) are locally weighted scatterplot smooth lines. Other, other type of keratoplasty. PKP, penetrating keratoplasty; ALTK/DALK, automated lamellar therapeutic keratoplasty/deep anterior lamellar keratoplasty; DSAEK, Descemet stripping automated endothelial keratoplasty; Other, other type of keratoplasty (see Figure 1).

The age-adjusted frequency of all keratoplasties increased from 1.4 to 3.3 per 100,000 in HUEH from 1995 to 2015. In primary PKP, locally weighted scatterplot smoothing showed a linearly decreasing trend in age-adjusted frequency, decreasing from 0.96 in 1995 to 0.46 in 2015 per 100,000. By contrast, the age-adjusted frequency of all PKP procedures, including regrafts, remained essentially stable until 2010 (Figs. 3A, B). In primary ALTK/DALK, a mild linear increase occurred from 1995 to 2005, followed by a plateau from 2005 to 2015. In primary DSAEK, there was an exponential increase from 2010 to 2015. The median age-adjusted frequencies for primary keratoplasties over the years 1995 to 2015 and over 5-year intervals are presented in Table 1.

**TABLE 1.** Age-Adjusted Frequency of Primary Keratoplasty per 100,000 in HUEH From 1995 to 2015 by Graft Type and 5-yr Periods

PKP	1995–2015	1995–2000	2001–2005	2006–2010	2011–2015
Median	0.89	1.16	0.77	0.89	0.79
Mean	0.94	1.18	0.75	1.03	0.75
Range	0.46–1.54	0.96–1.54	0.55–0.96	0.66–1.53	0.46–0.96

ALTK/DALK	1999–2015	1995–2000	2001–2005	2006–2010	2011–2015
Median	0.09	NA	0.06	0.15	0.13
Mean	0.14	NA	0.06	0.23	0.16
Range	0.02–0.43	NA	0.02–0.11	0.07–0.43	0.09–0.30

DSAEK	2006–2015	1995–2000	2001–2005	2006–2010	2011–2015
Median	0.29	NA	NA	0.20	0.35
Mean	0.39	NA	NA	0.22	0.56
Range	0.11–1.42	NA	NA	0.11–0.35	0.20–1.42

PKP, penetrating keratoplasty; ALTK/DALK, automated lamellar therapeutic keratoplasty/deep anterior lamellar keratoplasty;DSAEK, Descemet stripping automated endothelial keratoplasty.

### Patient Characteristics in HUEH

Of the 1574 patients, 51% were male. In primary keratoplasty, the male-to-female ratio was 1.1 for PKP, 1.9 for ALTK/DALK, and 0.5 for DSAEK.

Regarding primary keratoplasty, the median age of men and women was 48 and 67 years for PKP, 37 and 46 years for ALTK/DALK, and 66 and 68 years for DSAEK, respectively (Fig. 3D, see Supplemental Table 2, Supplemental Digital Content 5, http://links.lww.com/ICO/B364). In patients younger than 18 years, the median age was 10 years for PKP and 13 years for ALTK/DALK. No DSAEK were performed. Patient characteristics, including median age and number of grafts, by graft type, gender, and age groups, are displayed in Supplemental Digital Content 5, Supplemental Table 2, http://links.lww.com/ICO/B364.

**FIGURE 4.**
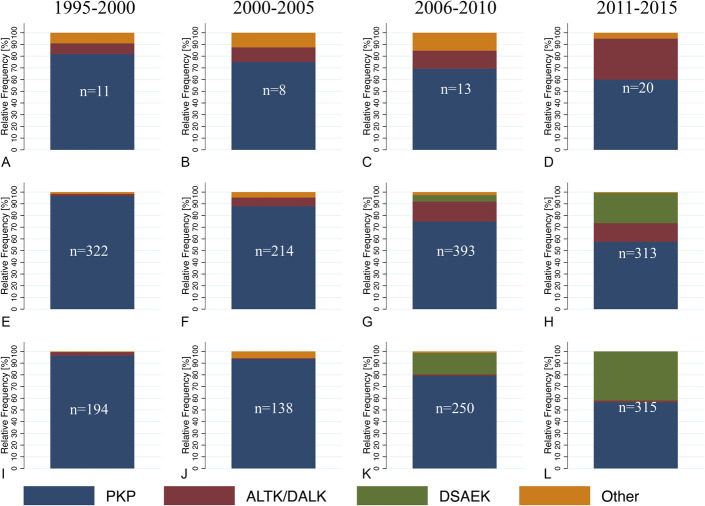
Relative frequency of grafts by age groups and time periods in Helsinki University Eye Hospital; (A-D) Age 0-18 years, (E-H) age 18-65 years, and (I-L) age over 65 years. Time periods 1995-2000, 2001-2005, 2006-2010 and 2011-2015 are displayed in columns. N, total number of grafts; PKP, penetrating keratoplasty; ALTK/DALK, automated lamellar therapeutic keratoplasty/deep anterior lamellar keratoplasty; DSAEK, Descemet stripping automated endothelial keratoplasty; Other, other type of keratoplasty (see Figure 1).

Divided by age groups 0 to 18 years, 18 to 65 years, and over 65 years and by periods of 1995 to 2000, 2001 to 2005, 2006 to 2010, and 2011 to 2015, a change in the type of keratoplasty performed was evident (Fig. 4). In patients younger than 18 years, the proportion of PKP decreased from 82% in 1995 to 2000 to 60% in 2011 to 2015 and that of ALTK/DALK increased from 9% to 35%. In patients aged 18 to 65 years, the proportion of PKP decreased from 97% in 1995 to 2000 to 58% in 2011 to 2015, that of ALTK/DALK increased from 2% to 16%, and that of DSAEK increased from 0% to 26%. In patients older than 65 years, the proportion of PKP decreased from 96% in 1995 to 2000 to 57% in 2011 to 2015 and that of DSAEK increased from 0% to 42%. In general, in all age groups, the relative proportion of PKP decreased, and in age groups 18 to 65 years and older than 65 years, the relative proportion of DSAEK increased.

**FIGURE 5.**
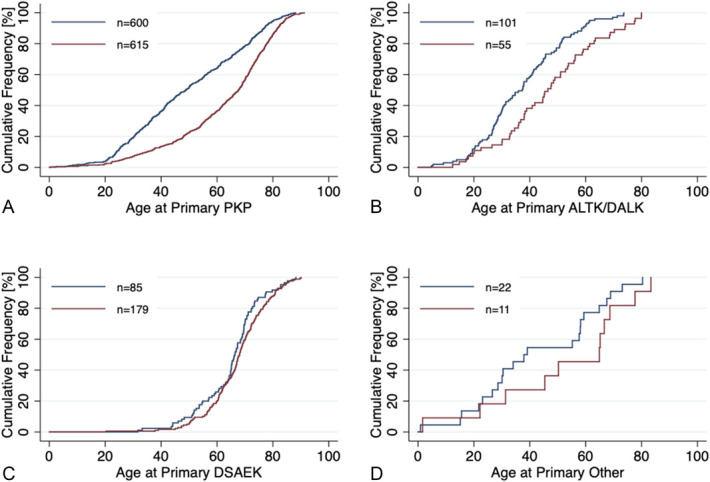
Cumulative frequency of age at the time of primary keratoplasty in 1995-2015 by type of graft in Helsinki University Eye Hospital. (A) PKP, (B) ALTK/DALK, (C) DSAEK, and (D) other keratoplasty procedures. Blue curve indicates male patients and red curve female patients. PKP, penetrating keratoplasty; ALTK/DALK, automated lamellar therapeutic keratoplasty/deep anterior lamellar keratoplasty; DSAEK, Descemet stripping automated endothelial keratoplasty; Other, other type of keratoplasty (see Figure 1).

In men, 50% of all primary grafts in PKP were performed by 48 years, in ALTK/DALK by 37 years, and in DSAEK by 66 years (Figs. 5A–C). The corresponding figures in women were 67, 46, and 68 years, respectively. Men underwent primary PKP (*P* = 0.0001, Kruskal–Wallis test) and ALTK/DALK (*P* = 0.0015) at an earlier age compared with women. In primary DSAEK, ages were comparable between sexes (*P* = 0.11).

### Regrafts in HUEH

**FIGURE 6.**
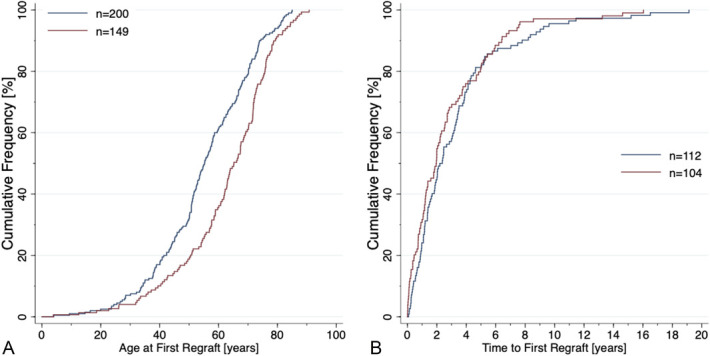
Cumulative frequency of age at the first regraft (A) and time to the first regraft by sex (B) in Helsinki University Eye Hospital from 1995 to 2015. Blue curve indicates male patients and red curve female patients.

Of all regrafts, 93% were PKP, and the male-to-female ratio was 1.5. In males, 50% of all first regrafts were performed by 55 years and in females by 65 years (Fig. 6A).

In 216 patients who received a primary graft from 1995 to 2015 and the first regraft in the same eye, the median interval was 2.0 years (range 0.02–16, Interquartile range (IQR) 0.9–3.8). Most, 93% of first regrafts, were PKP. Ten percent of first regrafts were performed within 4 months, 28% within 1 year, 50% within 2 years, 75% within 4 years, and 90% within 7 years (Fig. 6B). In men, 50% of the first regrafts were performed within 2.2 years and in women within 1.9 years (*P* = 0.17, Kruskal–Wallis test).

### Procurement of Corneal Transplants in HUEH from 2000 to 2015

Between 2000 and 2015, corneal transplants were procured by the HUEH Eye Bank or purchased at cost from other Finnish and Scandinavian Eye Banks, especially from Regea, the national Cell and Tissue Center, founded in 2005. The median annual number of grafts provided by the HUEH Eye Bank was 51 (range, 34–85), corresponding to a median percentage of 48% (range, 27%–84%) of all grafts (Fig. 2B).

Scandinavian Eye Banks provided transplants between 2000 and 2008; the highest proportion was in 2006, with 38% of all grafts. Other Finnish Eye Banks provided grafts from 2000 to 2007, with the highest proportion of 29% in 2003. In 2007, a total of 87 corneal grafts were purchased from the United States, funded with a donation from a nonprofit Finnish foundation, representing an annual proportion of 44%. Regea provided the first corneal grafts in 2007, with a proportion of 8%, increasing to 53% in 2015. From 2009 onward, all corneal grafts in HUEH were provided by its eye bank or Regea.

The increase in the annual number of keratoplasties was facilitated by an increasing number of corneal grafts provided by Regea (79 keratoplasties) to 2015 (181 keratoplasties) and HUEH Eye Bank from 2013 to 2015 (Figs. 2A, B).

### Population-Adjusted Frequency of All Keratoplasties in Finland From 2009 to 2015

The total number of keratoplasties performed in Finland increased from 176 in 2009 to 216 in 2015, and the proportion of HUEH increased from 69% to 84% of these. HUEH accounted for a median of 69% all keratoplasties in Finland from 2009 to 2015 (Table 2). During 2009 to 2015, Tampere University Eye Centre (TUEC) increased its proportion and accounted for a median of 9%. The median population-adjusted frequency of all keratoplasties was 3.2 per 100,000 from 2009 to 2015 (mean, 3.2; range, 2.5–3.9). Locally weighted scatterplot smoothing confirmed an increasing trend (Fig. 3C).

**TABLE 2.** Keratoplasties in Finland

Year	HUEH n (%)	TUEC n (%)	Other Centres* n (%)	Total n (%)
2009	121 (69)	18 (10)	37 (21)	176 (100)
2010	79 (58)	14 (10)	44 (32)	137 (100)
2011	84 (58)	8 (6)	53 (37)	145 (100)
2012	127 (65)	17 (9)	51 (26)	195 (100)
2013	128 (71)	7 (4)	46 (25)	181 (100)
2014	128 (74)	16 (9)	30 (17)	174 (100)
2015	181 (84)	12 (6)	23 (11)	216 (100)

* Department of Ophthalmology Oulu University Hospital, Department of Ophthalmology Kuopio University Hospital, Department of Ophthalmology Turku University Hospital.

HUEH, Helsinki University Eye Hospital; TUEC, Tampere University Eye Centre; TUEC, Tampere University Eye Centre.

## DISCUSSION

All residents of Finnish municipalities are entitled to public health care services. The municipalities are responsible for providing them by running 5 university hospitals and 15 central hospital districts, among other facilities. Among the university hospitals, HUEH is also the referral center for challenging patients in anterior segment surgery. Keratoplasties, with rare exceptions, are performed only in public university hospitals. We were able to identify 4 keratoplasties performed in the private sector in gathering our data.

In HUEH, the age-adjusted frequency of all keratoplasties increased over 2-fold during the study period. The relative proportion of primary PKP fell dramatically from 98% in 1995 to 1 quarter in 2015, and after 2006, DSAEK increased exponentially to nearly 70%. The introduction and rapid adoption of EK techniques resulted in increasing trend in age-adjusted frequency of primary grafts and a corresponding increase in the proportion of EK in regrafts. Earlier procurement of donor corneas from multiple different providers became centralized to the HUEH Eye Bank and Regea in 2009, which may have also facilitated the increase.

Regarding all keratoplasties in HUEH, the proportion of EK surpassed PKP in 2015, similar to the development in other European countries with public health care systems, such as Germany and France, in which the same happened in 2014 and, most likely, in 2016, respectively.^11,12^ EK became the most frequent keratoplasty already 2 years before the introduction of DMEK in HUEH, reflecting the excellent outcomes achieved with DSAEK.

Evolution of lamellar keratoplasty techniques resulted in different spectrums of procedures in different age groups. In regrafts, a change in the types of keratoplasties performed was also evident; during the study period, 90% of all regrafts were PKP, and in 2015, DSAEK accounted for 30% of regrafts.

In primary grafts, men underwent PKP and ALTK/DALK at a median of 18 and 9 years younger age than women, respectively. The median time interval to the first regraft was 2.0 years, with no difference between sexes. The male-to-female ratio was 1.0 in all grafts and 1.5 in regrafts. This higher male-to-female ratio is probably because men had more time to be at risk for a regraft.

At the end of the study period, HUEH accounted for 84% of all keratoplasties in Finland. After the study period, TUEC has increased its proportion of total keratoplasties because of the adoption of endothelial keratoplasty. In 2020, HUEH accounted for 64% and TUEC for 21% of all keratoplasties nationwide. In the year 2020, endothelial keratoplasty accounted for 78% of all keratoplasties in HUEH (39% were DSAEK and 39% were DMEK) and 94% in TUEC, all of which were DSAEK.

In Finland, the eye banks do not supply preloaded lamellar grafts. The corneal surgeon is responsible for preparing the corneal graft in the operating theater. This practice can elevate the bar to perform modern keratoplasty surgery in smaller university hospitals. In a recent retrospective comparative cohort study, clinical outcomes were similar at 1-year end point in patients with Fuchs endothelial dystrophy treated with DMEK using nonpreloaded and preloaded grafts.^22^

At the end of the study period, the population-adjusted frequency for total keratoplasties in Finland was 4 per 100,000. After population adjustment, 5 times more keratoplasties were performed in the United States and approximately 2 times more in Sweden, Germany, France, and New Zealand. Fewer keratoplasties were performed in South Korea, after population adjustment (see Supplemental Table 3, Supplemental Digital Content 6, http://links.lww.com/ICO/B365). EK techniques seem to be the most important factor in increasing the age-adjusted frequency of the total number of keratoplasties, and their wider adoption would be helpful in increasing the total frequency of keratoplasty in Finland.

Our study has several limitations. We were able to obtain the figures for total keratoplasties performed in Finland from 2009 to 2015, but not data about patient characteristics. Age adjustment for frequency of keratoplasties was therefore applied to HUEH data alone. Secondly, HUEH is a tertiary referral center in Finland, and compared with other Finnish centers, the patients referred to HUEH might represent more challenging cases, which can result in increased frequency of regrafts.

## Supplementary Material
