# Supplementary material for: Trends in Keratoplasty Procedures During 2 Decades in a Major Tertiary Referral Center in Finland: 1995 to 2015
Source: Cornea. 2022 Jan 25;42(1):36–43. doi: 10.1097/ICO.0000000000002990 (PMC9719831; doi:10.1097/ICO.0000000000002990)
Supplement: Supplementary file 1 [file cornea-42-36-s001.docx]

Supplementary Figure 1: (A) Number of primary keratoplasty procedures, (B) number, and (C) proportion of different type of primary keratoplasty procedures performed in Helsinki University Eye Hospital by year of grafting in 1995-2015. The red line in (A) is locally weighted scatterplot smoothing. PKP, penetrating keratoplasty; ALTK/DALK, automated lamellar therapeutic keratoplasty/deep anterior lamellar keratoplasty; DSAEK, Descemet stripping automated endothelial keratoplasty; Other, other type of keratoplasty.
