# Supplementary material for: Trends in Keratoplasty Procedures During 2 Decades in a Major Tertiary Referral Center in Finland: 1995 to 2015
Source: Cornea. 2022 Jan 25;42(1):36–43. doi: 10.1097/ICO.0000000000002990 (PMC9719831; doi:10.1097/ICO.0000000000002990)
Supplement: Supplementary file 3 [file cornea-42-36-s003.docx]

Supplementary Figure 3: Type of first regraft if primary graft performed in Helsinki University Eye Hospital from 1995 to 2015. PKP, penetrating keratoplasty; ALTK/DALK, automated lamellar therapeutic keratoplasty/deep anterior lamellar keratoplasty; DSAEK, Descemet stripping automated endothelial keratoplasty; Other, other type of keratoplasty.
