# Supplementary material for: Trends in Keratoplasty Procedures During 2 Decades in a Major Tertiary Referral Center in Finland: 1995 to 2015
Source: Cornea. 2022 Jan 25;42(1):36–43. doi: 10.1097/ICO.0000000000002990 (PMC9719831; doi:10.1097/ICO.0000000000002990)
Supplement: Supplementary file 4 [file cornea-42-36-s004.docx]

| **Supplementary Table 1:** All keratoplasties, primary grafts and regrafts performed in Helsinki University Eye Hospital by graft type, gender and age in 1995-2015. | | | | | | |
| --- | --- | --- | --- | --- | --- | --- |
|  | **Total** | **Male** | **Female** | **<18 years** | **18-65 years** | **>65 years** |
|  | **n (%)** | **n (%)** | **n (%)** | **n (%)** | **n (%)** | **n (%)** |
| **All Grafts** |  |  |  |  |  |  |
| PKP | 1701 (78) | 893 (80) | 808 (75) | 36 (69) | 973 (78) | 692 (77) |
| ALTK/DALK | 165 (8) | 109 (10) | 56 (5) | 11 (21) | 139 (11) | 15 (2) |
| DSAEK | 281 (13) | 88 (8) | 193 (18) | . | 103 (8) | 178 (20) |
| Other | 44 (2) | 29 (3) | 15 (1) | 5 (10) | 27 (2) | 12 (1) |
| Total | 2191 (100) | 1119 (100) | 1072 (100) | 52 (100) | 1242 (100) | 897 (100) |
| **Primary Grafts** | |  |  |  |  |  |
| PKP | 1215 (73) | 600 (74) | 615 (72) | 30 (67) | 673 (73) | 512 (73) |
| ALTK/DALK | 156 (9) | 101 (13) | 55 (6) | 11 (24) | 132 (14) | 13 (2) |
| DSAEK | 264 (16) | 85 (11) | 179 (21) | . | 97 (11) | 167 (24) |
| Other | 33 (2) | 22 (3) | 11 (1) | 4 (9) | 20 (2) | 9 (1) |
| Total | 1668 (100) | 808 (100) | 860 (100) | 45 (100) | 922 (100) | 701 (100) |
| **Regrafts** |  |  |  |  |  |  |
| PKP | 486 (93) | 293 (94) | 193 (91) | 6 (86) | 300 (94) | 180 (92) |
| ALTK/DALK | 9 (2) | 8 (3) | 1 (1) | . | 7 (2) | 2 (1) |
| DSAEK | 17 (3) | 3 (1) | 14 (7) | . | 6 (2) | 11 (6) |
| Other | 11 (2) | 7 (2) | 4 (2) | 1 (14) | 7 (2) | 3 (2) |
| Total | 523 (100) | 311 (100) | 212 (100) | 7 (100) | 320 (100) | 196 (100) |
| PKP, penetrating keratoplasty; ALTK/DALK, automated lamellar therapeutic keratoplasty/deep anterior lamellar keratoplasty; DSAEK, Descemet stripping automated endothelial keratoplasty. | | | | | | |
