# Supplementary material for: Trends in Keratoplasty Procedures During 2 Decades in a Major Tertiary Referral Center in Finland: 1995 to 2015
Source: Cornea. 2022 Jan 25;42(1):36–43. doi: 10.1097/ICO.0000000000002990 (PMC9719831; doi:10.1097/ICO.0000000000002990)
Supplement: Supplementary file 5 [file cornea-42-36-s005.docx]

| **Supplementary Table 2:** Patient characteristics by graft type, gender and age in Helsinki University Eye Hospital in 1995-2015. | | | | |  |  |  |  |  |  |  |  |  |  |  |
| --- | --- | --- | --- | --- | --- | --- | --- | --- | --- | --- | --- | --- | --- | --- | --- |
|  |  | **Age Male** |  |  | **Age Female** |  |  | **Age <18 years** |  |  | **Age 18-65 years** |  |  | **Age >65 years** |  |
| **All Grafts** | **Total** | **Median (Range)** | **Mean (SD)** | **Total** | **Median (Range)** | **Mean (SD)** | **Total** | **Median (Range)** | **Mean (SD)** | **Total** | **Median (Range)** | **Mean (SD)** | **Total** | **Median (Range)** | **Mean (SD)** |
| PKP | 893 | 53.0 (0.4-88) | 51.9 (18.8) | 808 | 66.6 (0.4-93) | 62.4 (17.6) | 36 | 10.2 (0.4-18) | 9.5 (4.8) | 973 | 47.7 (19-65) | 45.8 (12.8) | 692 | 74.2 (65-93) | 74.9 (6.1) |
| ALTK/DALK | 109 | 37.9 (5-85) | 38.8 (15.9) | 56 | 46.5 (12-80) | 46.8 (17.6) | 11 | 13.8 (5-18) | 13 (4.8) | 139 | 39.7 (18-63) | 40.2 (12.4) | 15 | 73.7 (69-85) | 74.2 (4.9) |
| DSAEK | 88 | 66.5 (32-88) | 65.1 (11.1) | 193 | 68.1 (20-90) | 67.5 (10.5) | . | . | . | 103 | 57.8 (20-65) | 55.9 (8.0) | 178 | 71.5 (65-90) | 73 (6.2) |
| Other | 29 | 56.5 (1-80) | 47.1 (22.6) | 15 | 50.3 (2-83) | 51.4 (21.8) | 5 | 15.1 (1-16) | 9.9 (7.9) | 27 | 45.4 (22-65) | 44.8 (14.5) | 12 | 73.4 (65-83) | 73 (5.7) |
| **Primary Grafts** |  |  |  |  |  |  |  |  |  |  |  |  |  |  |  |
| PKP | 600 | 48.9 (0.4-88) | 49.8 (20.0) | 615 | 67.7 (0.4-91) | 62.5 (18.0) | 30 | 10.2 (0.4-18) | 9.5 (5.0) | 673 | 44.5 (19-65) | 43.9 (13.1) | 512 | 74.7 (65-91) | 75.2 (6.0) |
| ALTK/DALK | 101 | 37.3 (5-74) | 37.5 (15.2) | 55 | 46.5 (12-80) | 46.8 (17.8) | 11 | 13.8 (5-18) | 13 (4.8) | 132 | 38.9 (18-63) | 39.9 (12.4) | 13 | 73.7 (69-80) | 73.8 (4.0) |
| DSAEK | 85 | 66.4 (32-88) | 65.1 (11.1) | 179 | 68 (20-90) | 67.5 (10.6) | . | . | . | 97 | 58.2 (20-65) | 55.9 (8.1) | 167 | 71.1 (65-90) | 73 (6.3) |
| Other | 22 | 38.5 (1-80) | 43 (22.1) | 11 | 64.9 (2-83) | 52.5 (25.2) | 4 | 8.4 (1-16) | 8.3 (8.1) | 20 | 38.5 (22-65) | 41.9 (15.3) | 9 | 68.9 (65-83) | 72.4 (6.6) |
| **Regrafts** |  |  |  |  |  |  |  |  |  |  |  |  |  |  |  |
| PKP | 293 | 57.2 (4-85) | 56.3 (14.9) | 193 | 63.5 (4-93) | 62 (16.5) | 6 | 10.3 (4-15) | 9.3 (4.5) | 300 | 52.7 (19-65) | 50.3 (10.9) | 180 | 72.8 (65-93) | 74 (6.2) |
| ALTK/DALK | 8 | 51.2 (24-85) | 54.6 (17.8) | 1 | . | 46.6 | . | . | . | 7 | 50.8 (24-60) | 47 (11.0) | 2 | 77.2 (69-85) | 77.2 (11.0) |
| DSAEK | 3 | 70.2 (54-75) | 66.3 (11.0) | 14 | 70.2 (49-82) | 67.1 (9.9) | . | . | . | 6 | 55.8 (49-62) | 55.5 (5.2) | 11 | 72.4 (67-82) | 73.1 (4.1) |
| Other | 7 | 61.6 (16-76) | 59.8 (20.8) | 4 | 45.1 (43-61) | 48.5 (8.5) | 1 | . | 16.4 | 7 | 56.5 (43-62) | 53.1 (8.5) | 3 | 74.9 (74-76) | 74.9 (1.2) |
| PKP, penetrating keratoplasty; ALTK/DALK, Automated Lamellar Therapeutic Keratoplasty/Deep Anterior Lamellar Keratoplasty; DSAEK, Descemet Stripping Automated Endothelial Keratoplasty; Other, other type of corneal graft. | | | | | | | | | | | | | | | |
