# Supplementary material for: Trends in Keratoplasty Procedures During 2 Decades in a Major Tertiary Referral Center in Finland: 1995 to 2015
Source: Cornea. 2022 Jan 25;42(1):36–43. doi: 10.1097/ICO.0000000000002990 (PMC9719831; doi:10.1097/ICO.0000000000002990)
Supplement: Supplementary file 6 [file cornea-42-36-s006.docx]

| **Supplementary Table 3:** Population-adjusted frequency of total keratoplasties per 100,000 population by country and year. | | | | | | | |
| --- | --- | --- | --- | --- | --- | --- | --- |
| **Year** | **Finland** | **USA (1)** | **Germany (2)** | **France (3)** | **Sweden (4)** | **South Korea (5)** | **New Zealand (6)** |
| 2009 | 3 | 14 | 5 | 5 |  | 2 |  |
| 2010 | 2 | 14 | 5 | 5 |  | 2 |  |
| 2011 | 2 | 14 | 6 | 5 |  | 2 |  |
| 2012 | 3 | 15 | 6 | 6 | 7 | 2 |  |
| 2013 | 3 | 15 | 7 | 6 | 5 | 2 | 7 |
| 2014 | 3 | 15 | 7 | 7 | 6 | 2 |  |
| 2015 | 3 |  | 8 | 7 | 6 | 2 |  |
| Table modified from references below. | | | |  |  |  |  |

1. Park CY, Lee JK, Gore PK, et al. Keratoplasty in the United States: A 10-year review from 2005 through 2014. Ophthalmology. 2015;122:2432-2442.

2. Flockerzi E, Maier P, Bohringer D, et al. Trends in corneal transplantation from 2001 to 2016 in Germany: A report of the DOG-Section Cornea and its Keratoplasty Registry. Am J Ophthalmol. 2018;188:91-98.

3. Ricouard F, Puyraveau M, Gain P, et al. Regional trends in corneal transplantation from 2004 to 2015 in France: a 12-year review on indications, technique and waiting period. Cell Tissue Bank. 2020;21:65-76.

4. Swedish Registry for Corneal Transplant Year Report 2012-2015 Swedish Registry for Corneal Transplant; 2012-2015 Available at: <http://www.cornea.nu/om-svenska-cornearegistret/arsrapporter/>. Accessed September 15, 2021

5. Shin KY, Lim DH, Han K, et al. Higher incidence of penetrating keratoplasty having effects on repeated keratoplasty in South Korea: A nationwide population-based study. PLoS One. 2020;15:e0235233.

6. Kim BZ, Meyer JJ, Brookes NH, et al. New Zealand trends in corneal transplantation over the 25 years 1991-2015. Br J Ophthalmol. 2017;101:834-838.
